# Should we suspect primary aldosteronism in patients with hypokalaemic rhabdomyolysis? A systematic review

**DOI:** 10.3389/fendo.2023.1257078

**Published:** 2023-09-22

**Authors:** Everardo Josué Díaz-López, Rocio Villar-Taibo, Gemma Rodriguez-Carnero, Antia Fernandez-Pombo, Roberto Garcia-Peino, Manuel Narciso Blanco-Freire, Alberto Pena-Dubra, Teresa Prado-Moraña, Irea- Fernández-Xove, Edurne Pérez-Béliz, Jose Manuel Cameselle-Teijeiro, Alvaro Hermida-Ameijeiras, Miguel Angel Martinez-Olmos

**Affiliations:** ^1^ Division of Endocrinology and Nutrition, University Clinical Hospital of Santiago de Compostela, Santiago de Compostela, Spain; ^2^ Unidad de Enfermedades Tiroideas e Metabólicas (UETeM)-Molecular Pathology Group. Department of Psychiatry, Radiology, Public Health, Nursing and Medicine, Health Research Institute of Santiago de Compostela (IDIS)-Center for Research in Molecular Medicine and Chronic Diseases (CIMUS), University of Santiago de Compostela, Santiago de Compostela, Spain; ^3^ Division of Epigenomics in Endocrinology and Nutrition Group-Health Research Institute of Santiago de Compostela (IDIS), University of Santiago de Compostela, Santiago de Compostela, Spain; ^4^ Division of Surgery, University Clinical Hospital of Santiago de Compostela, Santiago de Compostela, Spain; ^5^ Division of Pathology, University Clinical Hospital of Santiago de Compostela, Santiago de Compostela, Spain; ^6^ Medical Faculty, University of Santiago de Compostela, Santiago de Compostela, Spain; ^7^ Division of Internal Medicine, University Clinical Hospital of Santiago de Compostela, Santiago de Compostela, Spain; ^8^ Molecular Endocrinology Group-Health Research Institute of Santiago de Compostela (IDIS), University of Santiago de Compostela, Santiago de Compostela, Spain; ^9^ CIBER Pathophysiology of Obesity and Nutrition (CIBEROBN), Carlos III Health Institute, Madrid, Spain

**Keywords:** rhabdomyolysis, hypokalaemia, hypokalaemic rhabdomyolysis, primary aldosteronism, acute kidney injury

## Abstract

Severe hypokalaemia causing rhabdomyolysis (RML) in primary aldosteronism (PA) is a rare entity, and only a few cases have been reported over the last four decades. This systematic review and case report aims to gather all published data regarding a hypokalaemic RML as presentation of PA in order to contribute to the early diagnosis of this extremely rare presentation. With the use of PubMed Central, EMBASE, and Google Scholar, a thorough internet-based search of the literature was conducted to identify articles and cases with RML secondary to hypokalaemia due to PA between June 1976 and July 2023. The case study concerns a 68-year-old male patient with hypokalaemic RML at presentation of PA. In the systematic review of the literature, 37 cases of RML secondary to hypokalaemia due to PA have been reported to date. In summary, the median age was 47.5 years, the male/female ratio was 17/21, all patients presented symptoms (weakness and/or myalgia), all the patients were hypertensive, and only four patients had complications with acute kidney injury (AKI). Although PA rarely presents with RML, it should be suspected when marked hypokalaemia and hypertension are also present. Early detection and management are essential to reduce the frequency of manifestations such as AKI.

## 1 Introduction

Primary aldosteronism (PA), also known as Conn’s syndrome, is the most common and treatable cause of endocrine-related hypertension, with a prevalence of 5%–10% among patients with hypertension in primary care and 20% among patients with resistant hypertension (1). PA results from the excessive production of aldosterone independently of renin and angiotensin II and leads to increased renal tubular resorption of sodium and volume expansion, resulting in increased blood pressure and hypokalaemia.

Although hypokalaemia is common in this disorder, severe hypokalaemia causing rhabdomyolysis (RML) in PA is a rare entity, and only a few cases have been reported over the last four decades (2, 3).

RML is a syndrome characterised by the destruction of striated muscle, which triggers the consequent release of intracellular elements such as electrolytes, myoglobin, creatine kinase (CK), and aldolase (4, 5). The effects are recognised as a clinical syndrome of muscle injury that is associated with the development of myoglobinuria, electrolyte abnormalities, and often acute kidney injury (AKI) (6, 7). RML has been described in patients with electrolyte disorders and endocrine disorders such as hypothyroidism and hyperthyroidism, hyperaldosteronism, diabetes mellitus, and diabetic ketoacidosis (4). One of the most intriguing causes of RML is potassium deficiency (8). The mechanism of hypokalaemia-induced RML is still not clear. Profound hypokalaemia (serum potassium <2.5 mEq/L) might play an important role in muscle damage secondary to i) contraction of capillaries with reduced muscle blood supply and resulting in lysing muscle cells, ii) suppression of synthesis and storage of glycogen, and iii) deranged ion transport across the cell membrane (2, 9).

We present a case report and systematic review aimed at collecting and summarising the published data regarding hypokalaemic RML as a form of presentation of PA in order to contribute to the early diagnosis of this extremely rare presentation.

## 2 Case report

A 68-year-old man with a history of schizophrenia, type 2 diabetes mellitus, and uncontrolled hypertension of 8 years was evaluated in the emergency department due to a fall after an episode acute of muscle weakness in the lower limbs while walking.

He received home antihypertensive treatment with a beta-blocker, mineralocorticoid receptor antagonists (MRAs), and angiotensin-converting enzyme (ACE) inhibitors.

His blood pressure was 183/101 mmHg with a heart rate of 40 beats/minute. He presented a body mass index of 29.8 kg/m^2^. Laboratory tests showed severe hypokalaemia with serum potassium of 2.1 mmol/L, glycaemia of 459 mg/dL, creatinine of 2.1 mg/dL, myoglobinaemia with serum myoglobin of 10,453 ng/mL, and CK of 3,616 IU/L. An electrocardiogram presented a prolongation of the QT interval.

Four months earlier, he presented with serum potassium of 3.9 mmol/L and creatinine of 0.97 mg/dL.

His clinical records were reviewed, and 4 months earlier, his levels of potassium and creatinine were normal. He also presented a 10-mm right adrenal nodule on an abdominal computed tomography (CT) scan 11 years previously, which was unknown at the time of admission.

**Figure 1:**
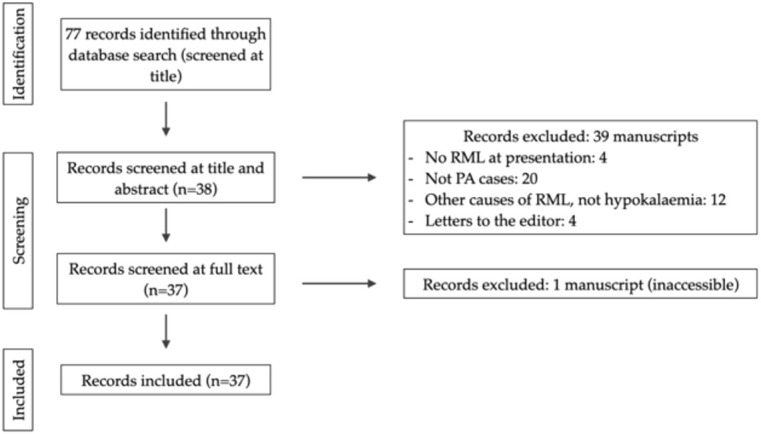
Preferred Reporting Items for Systematic Reviews and Meta-Analyses (PRISMA) flow chart for the identification of inclusion and exclusion of studies.

With the clinical suspicion of primary hyperaldosteronism, the patient underwent a hormonal analysis, which showed high levels of plasma aldosterone (188.0 ng/dL [upper limit of normal, 39.2 ng/dL]) and low plasma renin activity (0.18 ng/mL/h) with an aldosterone­to­renin ratio (ARR) of 1,074.3 ng/dL. His corresponding potassium was 4.1 mmol/L. Plasma cortisol, testosterone, dehydroepiandrosterone sulfate, 24-hour urine cortisol, and metanephrine levels were normal. A new adrenal CT was requested, observing a 27-mm nodule in the right adrenal gland, which showed attenuation of less than 10 Hounsfield units (**Figure 1**).

After stabilisation, the decision was taken to discharge the patient due to clinical improvement and normalisation of renal function, serum potassium, CK, and electrocardiogram alterations. Antihypertensives were changed to others that do not interfere with renin and aldosterone measurements in order to request a new hormonal study after 6 weeks.

During follow-up in the outpatient clinic, he presented an ARR of 866.7 ng/dL. Due to the presence of overt PA, no confirmatory studies were deemed necessary since this pattern is not observed in other entities (10).

**Figure 2:**
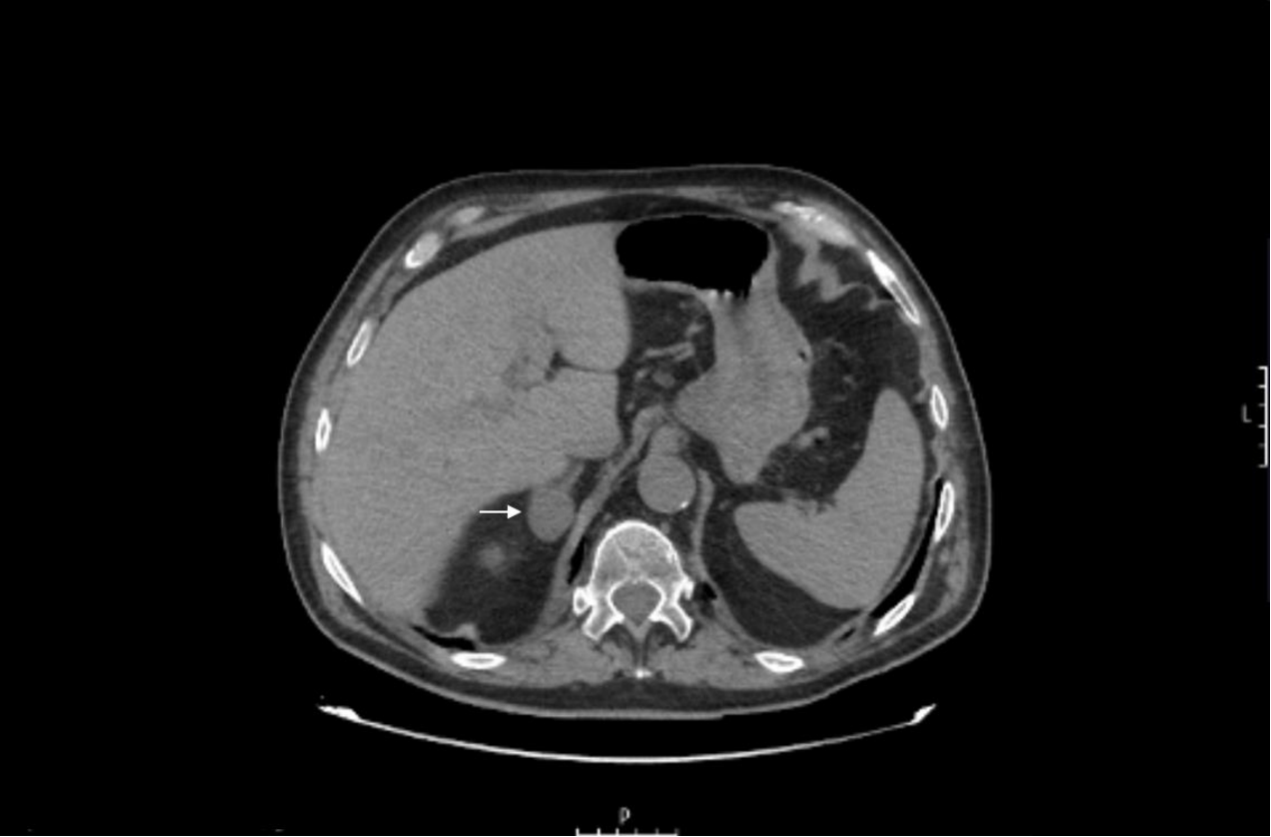
Adrenal computed tomography of the case study. White arrow showing a 27-mm nodule in the right adrenal gland.

**Figure 3:**
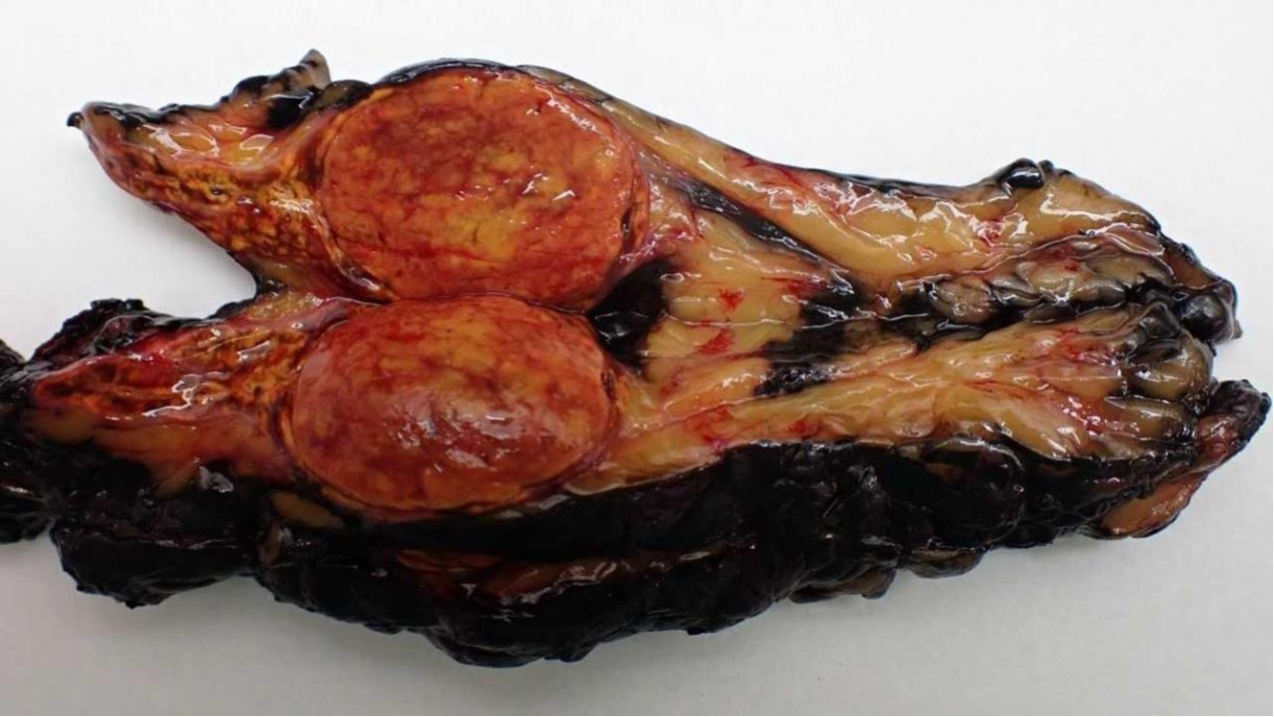
Aldosterone-producing adrenal cortical adenoma. The tumour is a solid, yellowish nodule that is well-demarcated by a thin rim of normal adrenal cortex.

The patient was evaluated by the Endocrine Tumour Committee at our hospital and scheduled for right posterior retroperitoneoscopic adrenalectomy. An adrenalectomy specimen with abundant adjacent adipose tissue was obtained, weighing 79.7 g and occupying a combined area of ​​10.8 × 4.2 cm. After the serial cuts, an adrenal gland with conventional characteristics was identified in an area of ​​up to 4.0 × 2.2 cm. The gland included a solid, well-delimited, yellowish nodule measuring 2.6 × 2.3 × 2.0 cm in diameter, compressing the residual adrenal tissue. A diagnosis of cortical adenoma was confirmed, pathologically consistent with aldosterone production. The adjacent adipose tissue did not present notable alterations (**Figure 2**). A 4.0-cm-diameter cortical adenoma was confirmed, pathologically consistent with aldosterone production. As an incidental finding, the tumour included spironolactone bodies (**Figure 3**).

Fifteen days after surgery, there was a clinical improvement in strength, and antihypertensive treatment (only with amlodipine 5 mg) was reduced due to good control of blood pressure. Potassium and aldosterone levels normalised without the need for oral potassium supplementation. The patient is currently fully recovered and is being monitored in primary care.

## 3 Research design and methods

### 3.1 Literature search and screening

The reported systematic review follows the Preferred Reporting Items for Systematic Reviews and Meta-Analyses (PRISMA) guidelines. The protocol was registered on PROSPERO International Prospective Register of Systematic Reviews, with registration ID CRD42023439678.

With the use of PubMed Central, EMBASE, and Google Scholar, a thorough internet-based search of the literature was carried out to identify articles and cases with RML secondary to hypokalaemia due to PA between June 17, 1976, and July 2, 2023. The search strategy was as follows: [(rhabdomyolysis) OR (hypokalaemia) OR (hypokalaemic rhabdomyolysis) OR (primary aldosteronism AND (hypokalaemia OR rhabdomyolysis)] OR (hypokalaemic rhabdomyolysis) OR (Conn’s syndrome).

### 3.2 Study selection and data management

Regarding study selection, data from the retrieved articles (title, authors, date of publication, journal, abstract, and keywords) were gathered from the major scientific platforms and transferred into Zotero (www.zotero.org) to identify and eliminate duplicate articles. Two independent reviewers (EJDL and RVT) initially screened the eligibility of studies based on the title/abstract content of each study identified. Studies that clearly did not satisfy the inclusion criteria were discarded with any disagreements being resolved by consensus or, if necessary, via consultation with a third reviewer (GRC). When no agreement was reached, a third reviewer (GRC) was involved to make the final decision.

All potentially eligible articles from this screening were downloaded as full text and distributed to the reviewers for verification that they fulfilled all the inclusion criteria and none of the exclusion criteria. Only cases or case series with hypokalaemic RML as presentation of PA were considered. Cases with no RML as presentation or other causes of RML not related to hypokalaemia were excluded. No restrictions on language or search period were included. In addition to the relevant articles, the articles referenced in the retrieved publications were also manually scrutinised.

General information from each article, such as first author or year of publication, was noted. Specific epidemiological data were also registered, including age and sex. The following clinical and biochemical variables were recorded: initial symptoms, antihypertensive drugs, presence of AKI, serum potassium (mmol/L), and creatinine (u/L). The treatment for PA and the anatomopathological diagnosis were also recorded.

Written informed consent was obtained from the patient for the publication of this article.

## 4 Results of the systematic review

**Figure 4:**
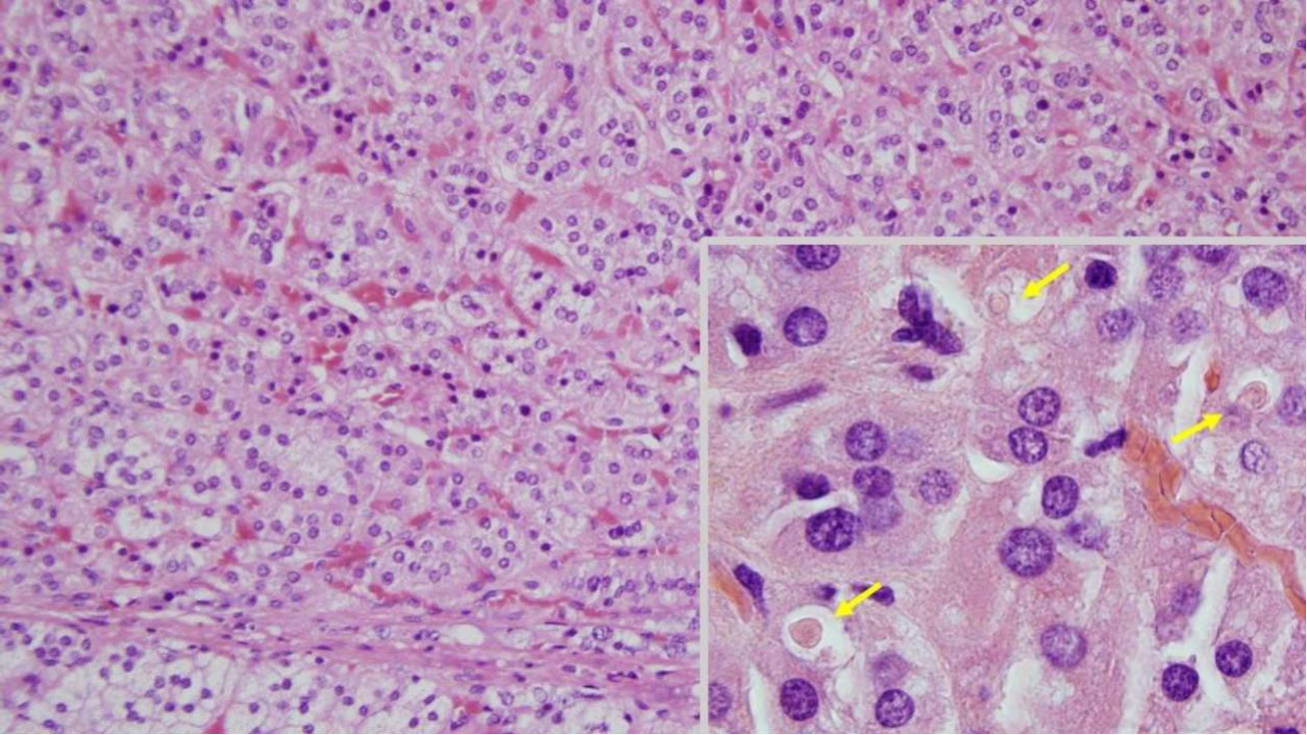
Adrenal histology of the case study. The adenoma is well-vascularised and composed of trabeculae of monomorphic cells with weakly eosinophilic cytoplasm, which contrasts with the clearer appearance of the cytoplasm of adjacent normal cells (bottom). At higher magnification (inset), spironolactone bodies can be seen (arrows); (hematoxylin and eosin, ×200; inset, ×1,000).

In the systematic review of the literature, 37 cases of RML secondary to hypokalaemia due to PA reported to date were identified. The flow chart of studies included is presented in **Figure 4**.

Clinical features, diagnosis data, treatment, and outcomes of all cases (including our patient) are presented in **Table 1**. In summary, the median age was 47.5 years, the male/female ratio was 17/21, all patients presented symptoms (weakness and/or myalgia), all the patients were hypertensive, and only four patients had complications with AKI.

**Table 1:** Clinical features, diagnosis data, treatment, and outcomes of all cases with hypokalaemic RML due to PA as a form of presentation.

Case/year (reference)	Country	Age (years)/sex	Initial symptoms	HT	Antihypertensive drugs	AKI	K^+^ (mmol/L)	CK (U/L)	Treatment	Subtype
1/1976 (11)	Canada	68/M	Weakness	Yes	HCTZ	No	0.6	3,250	UN	UN
2/1978 (12)	United States	49/M	Weakness	Yes	HCTZ	Yes	2.5	12,030	Surgical	Adenoma
3/1979 (13)	Japan	55/M	Weakness	Yes	Furosemide	No	1.8	2,000	Surgical	Adenoma
4/1981 (14)	United Kingdom	60/M	Weakness	Yes	Metoprolol	UN	1.4	36,000	UN	UN
5/1990 (15)	United States	32/F	Weakness	Yes	Beta-blocker, HCTZ	No	2.0	10,000	Surgical	Adenoma
6/1997 (16)	Canada	70/M	Weakness	Yes	Beta-blocker, amlodipine	Yes	1.8	2,800	Surgical	Adenoma
7/1998 (17)	France	64/M	Weakness	Yes	Enalapril, atenolol, nifedipine, HCTZ	No	1.3	2,997	Surgical	Adenoma
8/2002 (18)	Germany	30/F	Weakness	Yes	Fosinopril, amlodipine, HCTZ, furosemide	No	1.3	1,751	Surgical	Adenoma
9/2005 (19)	Turkey	44/F	Weakness	Yes	ACE inhibitor, verapamil	No	1.2	6,133	Surgical	Adenoma
10/2007 (20)	Poland	50/F	Weakness	Yes	Amlodipine	No	1.95	9,546	Surgical	Unilateral hyperplasia
11/2007 (21)	Greece	36/F	Weakness	Yes	Atenolol, chlorthalidone	Yes	2.2	2,860	Medical	NA
12/2008 (22)	Taiwan	28/F	Weakness	Yes	None	No	1.8	12,147	Surgical	Adenoma
13/2009 (23)	Germany	52/M	Weakness	Yes	Ramipril, Bisoprolol, HCTZ, clonidine	No	1.9	10,615	Surgical	Adenoma
14/2009 (24)	Greece	76/F	Weakness	Yes	Amlodipine	No	1.6	7,463	Surgical	Unilateral hyperplasia
15/2009 (25)	Turkey	14/F	None	Yes	None	UN	1.7	3,375	Surgical	Adenoma
16/2009 (26)	Japan	55/M	Weakness	Yes	Amlodipine, valsartan, HCTZ	No	1.4	15,760	Surgical	Adenoma
17/2009 (27)	Spain	46/F	Weakness and myalgias	Yes	None	No	1.3	21,000	Surgical	Adenoma
18/2012 (28)	Taiwan	49/F	Weakness	Yes	Amlodipine, valsartan	No	1.8	1,753	Medical	Bilateral nodular hyperplasia
19/2013 (29)	Austria	60/M	Weakness and myalgias	Yes	None	No	1.7	1,522	Surgical	Adenoma
20/2013 (30)	India	45/F	Weakness and myalgias	Yes	Nifedipine, atenolol, losartan	No	2.0	11,347	Surgical	Adenoma
21/2013 (31)	China	44/F	Weakness	Yes	UN	No	1.98	8,531	Surgical	Adenoma
22/2013 (31)	China	45/F	Weakness	Yes	Nitrendipine, captopril	No	1.38	4,907	Surgical	Adenoma
23/2015 (32)	China	38/M	Weakness and myalgias	Yes	Metoprolol, nifedipine	No	2.8	2,974	Surgical	Adenoma
24/2015 (33)	Japan	43/F	Weakness	Yes	Amlodipine, candesartan	No	1.8	6,929	Medical	Adenoma
25/2015 (2)	Italia	40/F	Weakness and myalgias	Yes	Irbesartan, HCTZ	No	1.66	9,122	Medical	Bilateral nodular hyperplasia
26/2015 (34)	Korea	54/M	Weakness	Yes	Felodipine, losartan, atenolol, HCTZ	No	2.0	2,982	Surgical	Adenoma
27/2017 (35)	Mexico	35/F	Weakness	Yes	Losartan, amlodipine	No	1.4	3,694	Surgical	Adenoma
28/2017 (36)	Albania	47/F	Weakness and myalgias	Yes	Amlodipine, valsartan	No	1.4	8,559	Surgical	NA
29/2018 (37)	Austria	55/F	Weakness and myalgias	Yes	Amlodipine, HCTZ, ARB, alpha- and beta-blockers	No	1.5	2,900	Surgical	Adenoma
30/2019 (38)	Taiwan	46/M	Weakness	Yes	Acebutolol, amlodipine, valsartan.	No	1.7	4,532	Medical	NA
31/2019 (39)	Russia	61/M	Weakness	Yes	ACE inhibitor, indapamide, bisoprolol	No	2.08	11,307	Medical	NA
32/2021 (3)	India	38/F	Weakness	Yes	MRA	No	1.9	1,015	Surgical	Adenoma
33/2021 (40)	Turkey	48/F	Weakness and myalgias	Yes	Valsartan, bisoprolol	No	1.3	14,248	Surgical	Adenoma
34/2021 (41)	Philippines	45/M	Weakness	Yes	Amlodipine	No	2.1	1,579	Surgical	Adenoma
35/2021 (9)	Taiwan	46/M	Weakness	Yes	Valsartan, HCTZ	No	1.9	1,626	Surgical	Adenoma
36/2021 (9)	Taiwan	53/M	Weakness and myalgias	Yes	Amlodipine, valsartan, HCTZ	No	2.1	1,593	Surgical	Adenoma
37/2022 (8)	China	65/F	Weakness and myalgias	No	None	No	1.8	18,370	Surgical	Adenoma
38/2023 (present case)	Spain	68/M	Weakness	Yes	Beta-blocker, ACE inhibitor, MRA	Yes	2.1	3,250	Surgical	Adenoma

M, male; F, female; UN, unknown; HCTZ, hydrochlorothiazide; NA, not applicable; HT, hypertension; AKI, acute kidney injury; ACE, angiotensin­converting enzyme; MRA, mineralocorticoid receptor antagonist; ARB, angiotensin receptor blocker; RML, rhabdomyolysis; PA, aldosteronism.

## 5 Discussion

Here, we present a patient who was suspected of having RML secondary to PA due to the presence of resistant hypertension and hypokalaemia, although this is a very rare presentation of PA, and it is more common for patients with RML to present hyperkalaemia due to the release of intracellular metabolites (potassium, phosphates, and urate) and intracellular proteins to the extracellular space and circulation (42).

The diagnosis of RML was based on the clinical presentation and the very high levels of CK and myoglobin. The myoglobinuria was detected by the presence of dark-coloured urine, with a positive urine dipstick test for blood without evidence of red blood cells on microscopy. This is a clue to the presence of RML, as myoglobin will also react with the orthotolidine test reagent (7).

A confirmatory test for PA was not required in this patient considering that current guidelines suggest bypassing such tests in patients with a particularly severe clinical phenotype (overt PA), i.e., patients with hypokalaemia, undetectable plasma renin, and plasma aldosterone concentrations higher than 20 ng/dL (555 pmol/L) (43).

In the systematic review, 21 out of 38 of the cases presented in women (55%), the majority of the patients (30/38) were younger than 60 years, and all the patients manifested symptoms (weakness and/or myalgias). In the Chinese biomedical literature database, 13 cases have been reported of PA being related to hypokalaemic RML. In this series, the epidemiological characteristics of the patients were similar, with a predominance of cases in women (69%), most of whom were under 60 years of age (84.6%). All 13 patients had a history of hypertension and weakness and/or myalgias. Twelve out of 13 patients had blood potassium lower than 2.5 mmol/L, 11 had adrenal adenoma, and none had renal failure (8). In a review of the English literature, 22 cases of PA related to hypokalaemic RML were summarised. Among these cases, nine patients were male and 13 were female (59%), five were 60 years of age or older and 17 were younger, 21 had symptoms of fatigue, and 20 had hypertension (2).

Regarding the levels of hypokalaemia related to RML occurrence, severe muscle weakness or RML usually occurs if serum potassium is below 2.5 mmol/L (44). In this review of cases, there are 35 patients who developed rhabdomyolysis with serum potassium below 2.1 mmol/L.

In the cases described, acute renal injury was very rare, being observed in only four patients (12, 16, 21). It was intensively managed with fluid therapy and intravenous potassium, with rapid clinical and analytical improvement. Intravenous fluids should be initiated as soon as possible, preferably within the first 6 hours after muscle injury (45).

In this review, 85% of the patients received surgery as the treatment for PA. The pathological diagnosis was adrenal adenomas in 93% of the cases. Of those who received definitive medical treatment (n = 5), three patients did not accept surgery, and two did not meet the criteria due to bilateral nodular hyperplasia. As an incidental finding, in our case, spironolactone bodies were detected. The incidence of spironolactone bodies within the adrenal gland in patients taking spironolactone or eplerenone is unknown. Patel et al., in a retrospective study, detected inclusions in only 33% of patients with PA treated with spironolactone and/or eplerenone (46). In this review, there were no cases other than our case describing this finding.

In conclusion, after reviewing the cases published in the literature, it can be concluded that hypokalaemic RML due to PA is a rare condition. It presents mostly in young patients (<60 years of age) with a certain predominance among women. It should be suspected in subjects with hypertension and mild hypokalaemia. Early diagnosis and management can lead to a better evolution of the patient, reducing the frequency of RML and complications such as AKI.

## Author contributions

ED-L: Conceptualization, Methodology, Supervision, Visualization, Writing – original draft, Writing – review & editing. RV-T: Conceptualization, Methodology, Supervision, Writing – review & editing. GR-C: Conceptualization, Writing – review & editing. AF-P: Supervision, Writing – review & editing. RG-P: Writing – review & editing. MB-F: Writing – review & editing. AP-D: Writing – review & editing. TP-M: Writing – review & editing. IF-X: Writing – review & editing. EP-B: Writing – review & editing. JC-T: Writing – review & editing. AH-A: Supervision, Writing – review & editing. MM-O: Writing – review & editing.
